# Neuroinflammation and Neutrophils: Modulation by Ouabain

**DOI:** 10.3389/fphar.2022.824907

**Published:** 2022-01-31

**Authors:** Jacqueline Alves Leite, Luiz Henrique Agra Cavalcante-Silva, Martina Raissa Ribeiro, Geovanni de Morais Lima, Cristoforo Scavone, Sandra Rodrigues-Mascarenhas

**Affiliations:** ^1^ Department of Pharmacology, Institute of Biomedical Science, Federal University of Goiás, Goiânia, Brazil; ^2^ Immunobiotechnology Laboratory, Biotechnology Center, Federal University of Paraíba, João Pessoa, Brazil; ^3^ Laboratory of Molecular Neuropharmacology, Department of Pharmacology, Institute of Biomedical Science, University of São Paulo, São Paulo, Brazil

**Keywords:** innate immunity, inflammation, ouabain, neuroimmune interactions, neuropharmacology

## Abstract

Cardiotonic steroids are natural compounds that present many physiological and pharmacological functions. They bind Na^+^/K^+^-ATPase (NKA) modifying cellular ion concentration and trigger cell signaling mechanisms without altering ion balance. These steroids are known to modulate some immune responses, including cytokine production, neutrophil migration, and inflammation (peripherally and in the nervous system). Inflammation can occur in response to homeostasis perturbations and is related to the development of many diseases, including immune-mediated diseases and neurodegenerative disorders. Considering the neutrophils role in the general neuroinflammatory response and that these cells can be modulated by cardiac steroids, this work aims to review the possible regulation of neutrophilic neuroinflammation by the cardiac steroid ouabain.

## Ouabain and Na^+^/K^+^-ATPase

Ouabain is a cardiotonic steroid identified by Hamlyn et al. (1991) in mammalian plasma. Studies have shown that ouabain can be produced by the adrenal gland, hypothalamus, and pituitary, being considered a hormone (Pamnani et al., 1981; Hamlyn et al., 1991; Ferrandi et al., 1997). In relation to the adrenal, ouabain synthesis appears to occur in the glomerulosa of the cortex (Laredo et al., 1995), and its release can be stimulated by two different hormones, angiotensin II, or adrenocorticotropic hormone (Laredo et al., 1994). The physiological levels of circulating ouabain in humans are approximately 0.2 nM and, in rodents, this value can reach 0.5 nM (Blaustein and Hamlyn, 2020).

Its receptor is the NKA, being the only established receptor for cardiotonic steroids such as ouabain, which interacts with amino acids located in the extracellular loops of the α subunit of the enzyme (Dvela et al., 2007). NKA is a membrane protein responsible for establishing and maintaining high K^+^ and low Na^+^ concentrations in the intracellular environment, in addition to maintaining cellular osmotic balance, the resting potential of most body tissues and the properties of excitable muscle and neural cells (Blanco and Mercer, 1998). The enzyme is composed of the α subunit, responsible for the catalytic activity and ion transport function, and the β subunit, which is necessary for the enzyme activity, regulating the fixation of the α subunit, and modulating the affinity for Na^+^ and K^+^ ions. There are four α isoforms, with α1 being ubiquitously expressed, while the α2–α4 isoforms present a more restricted cellular distribution (Markov et al., 2020). The modulating actions of this receptor affect both the cellular ionic balance, changing different cellular functions, such as cell migration (Ward and Becker, 1970), but also as an important signal transducer (Liu and Xie, 2010; Fan et al., 2017). At low concentrations (usually at nanomolar range), ouabain can promote conformational changes in NKA, without inhibiting the transport of sodium and potassium (Liu et al., 2000; Xie, 2003). This process leads to the activation/inhibition of many cell signaling proteins, including Src, MAPKs, and NF-κB (Cui and Xie, 2017).

Therapeutically, cardiotonic steroids are usually recommended for congestive heart failure, but only digoxin remains in use (Alpert, 2021). Despite this, ouabain presents many physiological and pharmacological effects that are more studied in the cardiovascular system, in the renal and brain tissues (reviewed in Blaustein and Hamlyn, 2020 and Bagrov et al., 2009). Other authors also suggest a repurposing of cardiac glycosides, including ouabain, for cancer therapeutics (Schneider et al., 2017; Matozzo et al., 2020; Du et al., 2021). Cardiac glycosides also modulate inflammation and autoimmune diseases (Škubník et al., 2021). Digoxin can inhibit transcriptional factor RORγt, thus inhibiting Th17 cells, a cell type involved in autoimmunity (Huh et al., 2011). Another cardiac glycoside, bufalin, inhibits the allergic inflammation by suppression of nuclear factor-kappa B activity (Zhakeer et al., 2017).

Regarding ouabain, many immune system cells can be modulated (Olej et al., 1994; Rodrigues-Mascarenhas et al., 2009). In thymocytes, precursor cells of T lymphocytes, ouabain inhibits cell proliferation (Szamel et al., 1981), induces the expression of CD69 (Rodrigues Mascarenhas et al., 2003) and the increase of free radicals (Smolyaninova et al., 2013). Also, in thymocytes, ouabain also reduces the activation of the mitogen-activated protein kinase (MAPK) p38 and the levels of the nuclear activating factor of T cells c1 (NFAT1c) (Rodrigues-Mascarenhas et al., 2008). Ouabain also inhibits lymphocyte mitosis. Furthermore, it was also evidenced that ouabain negatively modulates the number of B lymphocytes in the bone marrow, spleen, and peripheral blood (de Paiva et al., 2011), without, however, changing the levels of immunoglobulin G (IgG) and M (IgM). On the other hand, ouabain induces an increase in the number of B lymphocytes in the mesenteric lymph nodes, probably due to the reduction in the expression of the adhesion molecule CD62L and the chemokine receptor CXCR5 (da Silva et al., 2016).

Other immune system cells can also be modulated by ouabain. Nascimento et al. (2014) demonstrated that ouabain regulates the maturation of dendritic cells. In human monocytes, ouabain negatively regulates the expression of mCD14, a cell surface molecule involved in the response against Gram-negative bacteria, through the activation of the epidermal growth factor receptor (EGRF) and MAPK p38 (Valente et al., 2009). In addition, monocytes treated with ouabain have high levels of CD69, HLA DR, CD86, and CD80, molecules related to cell activation, in addition to increasing the phagocytic capacity of these cells (Teixeira and Rumjanek, 2014). Ouabain also inhibits, *in vitro*, the development of an inflammatory monocyte subtype (mCD14^+^CD16^+^) (Valente et al., 2009) and stimulates the production of cytokines such as IL-1α, IL-1β, TNF-α, IL- 6 and IL-10 (Foey et al., 1997; Matsumori et al., 1997; Teixeira and Rumjanek, 2014).

Moreover, ouabain can modulate inflammation and inflammatory cells (reviewed in Cavalcante-Silva et al., 2017). Dysregulated migration or activation of inflammatory cells, such as neutrophils are involved in the immunopathogenesis of many diseases (Leliefeld et al., 2016; Hellebrekers et al., 2018; Cavalcante-Silva et al., 2021b; Bautista-Becerril et al., 2021; Parackova et al., 2021). Thus, modulating these cells can be a therapeutic approach for inflammatory diseases. The ouabain effect on neutrophil during peripheral and neuro inflammation will be discussed below.

## Neuroinflammation and Neutrophils

For a long time, the central nervous system (CNS) was recognized as a “privileged immune” organ due to the presence of the blood-brain barrier (BBB), which was previously considered to be almost impenetrable. Nevertheless, several studies have pointed out the flexibility in the BBB in response to inflammatory stimuli, which can generate a process known as neuroinflammation (Kanashiro et al., 2020). Neuroinflammation is related to the emergence of neurodegenerative diseases, such as Parkinson’s disease, Alzheimer’s disease (AD) and cerebral ischemia as well as its association with neuropsychiatric disorders. Thus, neuroinflammation has been studied as an important therapeutic target for the treatment of neurodegenerative and neuropsychiatric diseases (Akira et al., 2006).

Inflammation in the CNS is orchestrated by resident immune cells such as astrocytes and microglia (Doty et al., 2015), as well as by the migration of monocytes and lymphocytes through the BBB. (Hawkes and McLaurin, 2009; Lampron et al., 2013). Several studies have pointed out the relevance of neutrophils in chronic neuroinflammatory diseases such as AD, but their role still needs to be better elucidated. Neutrophil recruitment can cause neuronal damage and cognitive decline in AD; however, some granular proteins, such as CAP37, neutrophil elastase and cathepsin G can promote Aβ cleavage, facilitating Aβ clearance, preventing the formation of pathogenic aggregates (reviewed in Stock et al., 2018).

CNS cells, such as astrocytes, release several chemokines dependent on the IL-17 pathway, such as CXCL5, CXCL2, and CXCL1, which promote neutrophil migration to the CNS. Furthermore, IL-17^−/−^ mice have a lower number of neutrophils infiltrating the CNS, but the same was not observed in the spinal cord, as neutrophil migration to the spinal cord appears to be regulated by IFN-γ (Christy et al., 2013; Simmons et al., 2014; Pierson et al., 2018). On the other hand, in chronic neurodegenerative diseases such as AD, neutrophil migration appears to be orchestrated by the chemokines CXCL12 and CCL2 as well as by the LFA-1 integrin (Zenaro et al., 2015). Interestingly, studies in mice have shown that blocking the LFA-1 integrin resulted in the inhibition of neutrophil migration, as well as the activation of microglia, resulting in reduced cognitive deficit in AD (Zenaro et al., 2015).

Neutrophil infiltration in post-ischemic inflammation has been associated with exacerbation of brain injury and neuronal damage due to release of pro-inflammatory cytokines, production of reactive oxygen species and reactive nitrogen species, and (neutrophil extracellular traps) NETs release. Furthermore, these neutrophils have a hypersegmented characteristic and their migration is dependent on the CXCR2 neutrophil-specific chemokine receptor (Herz et al., 2015; Neumann et al., 2015). Additionally, it was observed that blocking through a neutralizing antiserum or selective pharmacological inhibitor for CXCR2 prevented the recruitment of neutrophils to the brain of hyperlipidemic mice ApoE^−/−^ mice (Herz et al., 2015). Furthermore, it was observed that polymorphonuclear cells develop direct neurotoxicity through the secretion of TNF-alpha, matrix metalloproteinase 9, as well as through heterocellular contact, which may thus contribute to secondary damage after brain injury, such as cognitive impairment (Dinkel et al., 2004; Nguyen et al., 2007). Another important point observed in spinal cord injury models is the participation of NF-κB signaling in neutrophil activation and infiltration, since IκB kinase (IKK)-β conditional knockout mice showed lower secretion of CXCL1 and the consequent neutrophil infiltration resulted in less neuronal damage, neuroinflammation and improvement in motor function (Kang et al., 2011). Furthermore, Zenaro et al. showed that neutrophil infiltration plays an important role in microglia activation, accumulation of abnormal Aβ and tau, synaptic dysfunction, and memory decline in the neuroinflammation observed in AD (Zenaro et al., 2015).

On the other hand, the neuroprotective role for neutrophils in post-ischemic inflammation has been observed by different works. In an *in vitro* study conducted by Hou et al. (2019) it was observed that while N1 neutrophils decreased neuronal viability, N2 polarization promoted an improvement in neuronal viability against oxygen glucosedeprivation/re-oxygenation-induced injury, in cultured cortical neurons (Hou et al., 2019). In addition, using an *in vivo* model of injury induced by transient occlusion of the middle cerebral artery, Hou et al. (2019) showed that rats spontaneously regenerate over time, and that there is a negative correlation between the proportion of N2 neutrophils and the number of degenerating neurons, in the ipsilateral brain parenchyma (Hou et al., 2019). Furthermore, a study conducted in mice demonstrated that rosiglitazone, a peroxisome proliferator-activated receptor-γ (PPARy) agonist, increased the infiltration of N2-type neutrophils, culminating in a neuroprotective effect after stroke. Interestingly, the neuroprotective effect of the PPARγ agonist was reversed after neutrophil depletion, demonstrating their importance in neuroprotection (Cuartero et al., 2013). In another study, García-Culebras et al. (2019) demonstrated that mice deficient in TLR4 have a greater polarization for the N2 profile, resulting in a smaller infarct volume and neuroprotection, elucidating a role of TLR4 signaling for neutrophil polarization during the cerebral ischemia (García-Culebras et al., 2019). In addition, Sas et al. showed a CD14^+^ Ly6G^low^ granulocyte with features of an immature neutrophil with neuroregenerative and neuroprotective properties in models of optic nerve and spinal cord injury, resulting from the secretion of NGF and IGF-1 growth factors by CD14^+^ Ly6G^low^ cells (Sas et al., 2020).

**FIGURE 1:**
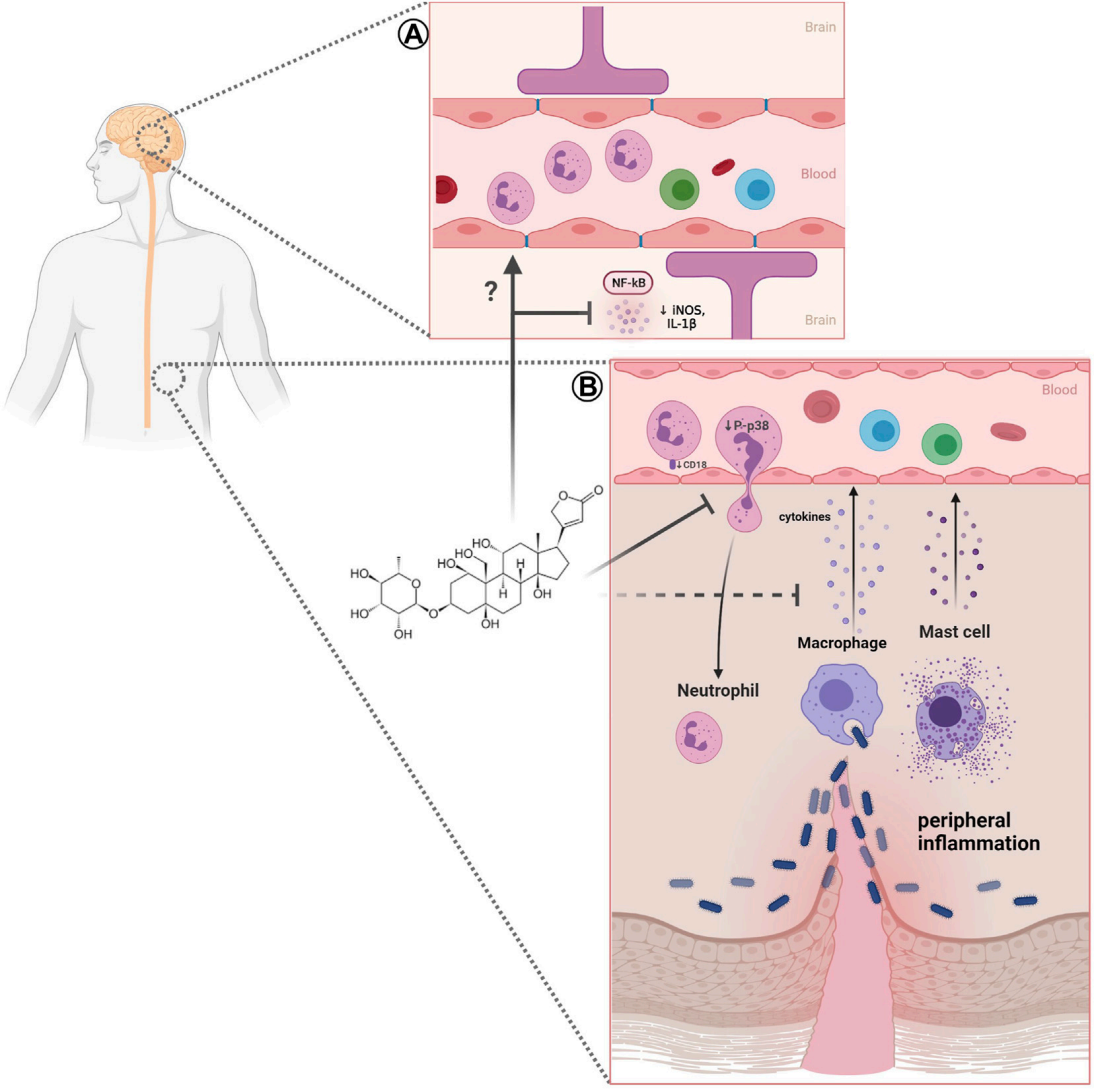
Effect of ouabain on neutrophils in peripheral and neuroinflammation. **(A)** In neuroinflammation ouabain can reduces NF-κB and iNOS activation and inflammatory cytokines (IL-1), however, the effects of this steroid in neutrophils remains to be elucidated. **(B)** Impairment of neutrophil migration caused by ouabain may involve reduction of adhesion molecule CD18, P-p38 MAPK, and decrease of inflammatory cytokines. Created with BioRender.com.

The different findings indicate that neutrophils are highly responsive to CNS lesions and may influence the process of neuroinflammation and neurodegeneration, as well as neuroprotection, affecting the development of neurodegenerative processes. Considering the development of neuroinflammation, ouabain emerge as a possible player in the regulation of this process (Figure 1A), as several studies have demonstrated the importance of digitalis and NKA in neuroinflammatory regulation (reviewed in Orellana et al., 2016).

Studies have demonstrated an *in vivo* and *in vitro* neuroprotective activity of ouabain. Kinoshita et al. (2014) have observed *in vivo* that ouabain has a protective effect against LPS-induced neuroinflammation in the rat hippocampus, through a reduction in the activation and consequent translocation of nuclear factor kappa B (NF-κB), leading to reduction in the expression of iNOS and IL-1β. Furthermore, it was observed that the administration of ouabain reduced the activation of astrocytes, in the dentate gyrus, through a reduction in the expression of glial fibrillary acidic protein (GFAP) (Kinoshita et al., 2014). In addition, *in vitro* studies of LPS-stimulated astrocytes found that treatment with ouabain reduced the release of IL-1β (Forshammar et al., 2011). On the other hand, an *in vitro* study using LPS-stimulated rat microglial cell culture, it was shown that treatment with Ouabain did not alter the release of TNF-α and IL-1β, thus suggesting a lack of modulating effect of ouabain on this cell type (Forshammar et al., 2013). Additionally, in a recently published study, Mázala-de-Oliveira et al. (2021) have shown that nanomolar concentrations of ouabain reduced the expression of inflammatory receptors, such as TNFR1, TLR4 and CD14 of rat retinal ganglion cells culture after optic nerve axotomy, in all tested periods. It was also observed that ouabain produced an increased survival of retinal ganglion cells after 48h, and the mechanism was dependent on autophagy, since the use of 3-methyladenine, an autophagy inhibitor, lead to a complete inhibition of the neuroprotective role of ouabain (Mázala-de-Oliveira et al., 2021).

On the other hand, it was shown that α2-NKA knockdown in superoxide dismutase 1 (SOD1) mutant astrocytes protected motor neurons from degeneration (Gallardo et al., 2014). Furthermore, it was observed that the silencing of α2-NKA in primary culture of glial cells from mice promoted a lower responsiveness to the stimulus with LPS, by reducing the production of TNF-α, as well as the activation of ERK and NF-kB, suggesting the participation of α2-NKA in the modulation of LPS-induced neuroinflammation (Kinoshita et al., 2017). Ouabain, *in vitro*, protected motor neurons from degeneration induced by mutant SOD1 astrocytes (Gallardo et al., 2014). Heterozygous KI mice, carrying the G301R disease mutation (α2^+/G301R^ mice), exhibited familial hemiplegic migraine type 2 (FHM2)-related phenotypes, including mood depression and obsessive-compulsive disorder (OCD)-like symptoms (Isaksen and Lykke-Hartmann, 2016). Surprisingly, when subjected to spinal cord injury, α2^+/G301R^ mice show better functional recovery and decreased lesion volume compared to littered controls (α2^+/+^). These phenotypes were associated with alterations in pro and anti-inflammatory cytokines levels, such as IL-6, TNF, and IL-10 (Ellman et al., 2017). Furthermore, it was observed that α2^+/G301R^ mice showed a reduction in the systemic levels of the proinflammatory cytokines TNF-α, IL-6 and IL-1β after LPS administration, as well as a reduction in the hypothermic and neuroinflammatory response in the hippocampus and hypothalamus (Leite et al., 2020).

Additionally, peripheral inflammation can also be modulated by ouabain. This steroid can alter vascular permeability induced by different inflammatory agents. In the sheep skin and pleural cavity, ouabain reduces vascular permeability caused by turpentine, an irritant agent (Lancaster and Vegad, 1967). Additionally, in mice peritoneal cavity this steroid decreases vascular permeability induced by zymosan, a fungal wall component (Leite et al., 2015). Also in mice, ouabain can inhibits paw edema, a cardinal signal resulting of increased vascular permeability, induced by carrageenan, compound 48/80, zymosan, prostaglandin E2, and bradykinin (de Vasconcelos et al., 2011). This effect of ouabain in vascular parameters could be related to its effects on histamine (Okazaki et al., 1976) and/or cytokines release (Leite et al., 2015). Indeed, it has been reported that ouabain decreases levels of cytokines TNF-α, IL-1β (Leite et al., 2015), and IFN-γ (Jacob et al., 2013) in peritoneal inflammation.

It is noteworthy that many studies related that ouabain possesses a proinflammatory effect and facilitates immune cell migration (Kennedy et al., 2013; Gonçalves-de-Albuquerque et al., 2014; Chen et al., 2017). The possible dual effect of ouabain on inflammation may be due to different animal species studied (i.e., BALB/c, Swiss, or C57BL/6 mice; rats; humans) since it could impact NKA sensitivity to ouabain. Moreover, administration route used, presence of a previous inflammatory stimulus, or even different concentrations of this steroid leads to different outcomes. In fact, high levels of ouabain could cause an immune system activation and promote a pathological inflammatory response (Blaustein and Hamlyn, 2020).

## Neutrophils: Modulation by Ouabain

During inflammation, endothelial cell activation by cytokines induces vascular permeability and enables migration of immune cells into tissues. Usually, neutrophils are the first immune cell to reach inflamed tissue (Liew and Kubes, 2019). Neutrophils are polymorphonuclear segmented cells with antimicrobial properties (Burn et al., 2021). However, these cells present many other immune functions and participate in the stimulation of adaptive immune responses (Minns et al., 2019), in the resolution of inflammation (Jones et al., 2016) and healing (Phillipson and Kubes, 2019), and have anti or pro-tumor activity (Mishalian et al., 2017; Ocana et al., 2017; Burn et al., 2021). Outside of neutrophils’ essential role in immune system homeostasis, they are also involved in autoimmune and inflammatory diseases, such as arthritis (O’Neil and Kaplan, 2019) and COVID-19 (Cavalcante-Silva et al., 2021b). In these pathological conditions, neutrophils may have a dysregulated migration or activation (Hidalgo et al., 2019).

Neutrophil migration requires the interaction between adhesion molecules present on neutrophils and vascular endothelium (Nourshargh and Alon, 2014). Classic neutrophil recruitment involves different steps such as capture, rolling, adhesion, crawling, and subsequent transmigration towards inflammatory signals (Kolaczkowska and Kubes, 2013). Several works have demonstrated the usually low concentrations of ouabain impairs mice neutrophil migration into different tissues. Leite et al. (2015) showed that ouabain reduces neutrophil migration into the peritoneal cavity induced zymosan. Similar findings were obtained using *L*. *amazonensis* as an inflammatory stimulus (Jacob et al., 2013). Other works provide evidence that ouabain also inhibits neutrophil transmigration into lung tissue in mice models of inflammatory allergy (Galvão et al., 2017) and acute pulmonary injury (Wang et al., 2018). *In vitro* studies also presented the inhibitory effect of ouabain on rabbit (Ward and Becker, 1970), human (Ray and Samanta, 1997), and mice neutrophil chemotaxis (Cavalcante-Silva et al., 2021a). The cardiotonic steroid marinobufagenin also reduces neutrophil migration during inflammation, corroborating the ouabain effect (Carvalho et al., 2019) (Figure 1B).

The exact mechanism of action of ouabain in impairment neutrophil migration remains to be fully elucidated, however, some evidence is emerging. Chemoattractant gradients trigger neutrophils intracellular signaling that guides these cells towards inflammatory tissues. The MAPK signaling mediates neutrophils chemotaxis (Liew and Kubes, 2019). It was observed that ouabain reduces p38 phosphorylation, but not ERK activation in mice neutrophils (Cavalcante-Silva L. H. A. et al., 2021). Neutrophil’s receptors, including adhesion molecules, can be regulated by p38 MAPK-dependent signaling (Kim and Haynes, 2013). Indeed, ouabain can reduce α (Ninsontia and Chanvorachote, 2014) and β (Cavalcante-Silva et al., 2020) integrins in different types of cells, nevertheless the real impact of this effects in neutrophil migration should still be addressed. On the other hand, ouabain does not reduce the chemokine receptor CXCR2 expression in mice neutrophils or modulates the levels of its ligands CXCL1 (Cavalcante-Silva et al., 2020). However, in human neutrophils, ouabain interferes with chemokine receptor (CXCR1/2) recycling, which in turns decreases neutrophil migration (Ray and Samanta, 1997).

Additionally, in models of peritoneal (Leite et al., 2015) and pulmonary (Wang et al., 2018) inflammation, ouabain also inhibits NF-κB pathway. The activation of this transcription factor is associated with release of proinflammatory cytokines, which in turn stimulates endothelial and immune cells (Netea et al., 2017). In fact, ouabain reduces TNF-α and IL-β release (Leite et al., 2015), therefore this may be associated with impaired migration of neutrophils. Moreover, in A549 cells, this steroid decreases the TNF-α-induced expression of ICAM-1, an adhesion molecule that binds integrin (Takada et al., 2009).

The mechanisms used by neutrophils during an immune response include phagocytosis, NETs, formation of reactive oxygen species and release of microbicidal molecules contained in cytoplasmic granules (i.e., myeloperoxidase, neutrophilic elastase, and others) (Yang et al., 2017). Neutrophils also produce different cytokines (eg, IL-1Ra, IL-12, IL-23, TNF-α, G-CSF, among others) and chemokines (eg, CXCL-1, CCL-20, CCL-2, among others) that modulate the immune response (Mantovani et al., 2011; Yang et al., 2017; Burn et al., 2021). In human neutrophils, it has been shown that ouabain at 100 nM induces DNA release, without promote necrosis, suggesting NETs release (Silva et al., 2021). Additionally, in rat neutrophils, ouabain reduces generation of free radical induced by NO donors, this effect could be related to membrane depolarization (Patel et al., 2009).

Although studies have shown that ouabain interferes with neutrophil infiltration induced by different stimuli in the periphery, such as concanavalin A (de Vasconcelos et al., 2011), Zimosan (Leite et al., 2015), ovalbumin (Galvão et al., 2017) and *Leishmania amazonensis* (Jacob et al., 2013), as well as the activation of these cells, since ouabain modulate the generation of free radicals induced by nitric oxide donors (Patel et al., 2009) and the release of NETs (Silva et al., al, 2021), studies lack data about ouabain modulation on neutrophils in the neuroinflammation; however, a study observed the presence of neutrophils in a model of ouabain-induced injury in zebrafish. Mitchell et al. (2018) observed that the retinal lesion resulting from the administration of ouabain was accompanied by an early leukocyte infiltration, followed by a period in which there was proliferation of immune cells, possibly from the resident microglia and macrophages derived from extra-retinal regions. Furthermore, the presence of neutrophils was observed in the vitreous leukocyte population, identified by the expression of myeloid-specific peroxidase (mpx) +, supporting that retinal injury induced by ouabain is accompanied by an early migration of leukocytes from the bloodstream. After 24, 48 and 72 h of ouabain administration, very few mpx + neutrophils were identified, suggesting that there was no significant increase in the number of neutrophils at these times (Mitchell et al., 2018).

Despite there is no compelling data on the effects of ouabain and its receptor, NKA, on neutrophil dynamics in neuroinflammatory diseases, robust studies point to the importance of ouabain-NKA signaling in neuroinflammation (reviewed in Orellana et al., 2016; Leite et al., 2020). In addition, the immunomodulatory role of ouabain in neutrophil dynamics has been observed in classical models of peripheral inflammation (Leite et al., 2015; Cavalcante-Silva et al., 2020). These data together suggest that ouabain-NKA signaling may be an important marker to be investigated in neutrophil dynamics in neuroinflammatory diseases, thus favoring a better understanding of the pathophysiology involved in the progression of these diseases, as well as aiding in the discovery of new strategies for neurodegenerative diseases.
